# Cooper Test Provides Better Half-Marathon Performance Prediction in Recreational Runners Than Laboratory Tests

**DOI:** 10.3389/fphys.2019.01349

**Published:** 2019-11-05

**Authors:** José Ramón Alvero-Cruz, Elvis A. Carnero, Manuel Avelino Giráldez García, Fernando Alacid, Thomas Rosemann, Pantelis T. Nikolaidis, Beat Knechtle

**Affiliations:** ^1^Faculty of Medicine, Andalucía TECH, University of Málaga, Málaga, Spain; ^2^Florida Hospital Sanford, Translational Research Institute for Diabetes and Metabolism, Burnham Prebys Medical Discovery Institute, Orlando, FL, United States; ^3^Faculty of Sports Science and Physical Education, University of A Coruña, A Coruña, Spain; ^4^Department of Education, Health Research Centre, University of Almería, Almería, Spain; ^5^Institute of Primary Care, University of Zurich, Zurich, Switzerland; ^6^Exercise Physiology Laboratory, Nikaia, Greece; ^7^Medbase St. Gallen am Vadianplatz, St. Gallen, Switzerland

**Keywords:** prediction equations, comparison performance methods, long-distance runners, field test, laboratory test

## Abstract

This study compared the ability to predict performance in half-marathon races through physiological variables obtained in a laboratory test and performance variables obtained in the Cooper field test. Twenty-three participants (age: 41.6 ± 7.6 years, weight: 70.4 ± 8.1 kg, and height: 172.5 ± 6.3 cm) underwent body composition assessment and performed a maximum incremental graded exercise laboratory test to evaluate maximum aerobic power and associated cardiorespiratory and metabolic variables. Cooper’s original protocol was performed on an athletic track and the variables recorded were covered distance, rating of perceived exertion, and maximum heart rate. The week following the Cooper test, all participants completed a half-marathon race at the maximum possible speed. The associations between the laboratory and field tests and the final time of the test were used to select the predictive variables included in a stepwise multiple regression analysis, which used the race time in the half marathon as the dependent variable and the laboratory variables or field tests as independent variables. Subsequently, a concordance analysis was carried out between the estimated and actual times through the Bland-Altman procedure. Significant correlations were found between the time in the half marathon and the distance in the Cooper test (*r* = −0.93; *p* < 0.001), body weight (*r* = 0.40; *p* < 0.04), velocity at ventilatory threshold 1, (*r* = −0.72; *p* < 0.0001), speed reached at maximum oxygen consumption (*v*VO_2_max), (*r* = −0.84; *p* < 0.0001), oxygen consumption at ventilatory threshold 2 (VO_2_VT2) (*r* = −0.79; *p* < 0.0001), and VO_2_max (*r* = −0.64; *p* < 0.05). The distance covered in the Cooper test was the best predictor of time in the half-marathon, and might predicted by the equation: Race time (min) = 201.26 – 0.03433 (Cooper test in m) (*R*^2^ = 0.873, SEE: 3.78 min). In the laboratory model, *v*VO2max, and body weight presented an *R*^2^ = 0.77, SEE 5.28 min. predicted by equation: Race time (min) = 156.7177 – 4.7194 (*v*VO_2_max) – 0.3435 (Weight). Concordance analysis showed no differences between the times predicted in the models the and actual times. The data indicated a high predictive power of half marathon race time both from the distance in the Cooper test and *v*VO2max in the laboratory. However, the variable associated with the Cooper test had better predictive ability than the treadmill test variables. Finally, it is important to note that these data may only be extrapolated to recreational male runners.

## Introduction

The number of half-marathon runners has increased steadily over the past ten years. As an example, in the United States the number has doubled in just a decade, reaching two million in 2013. Similarly, in Europe, it is estimated that there are around 50 million people who are regular long-distance runners (Scheerder et al., 2015). Furthermore, in Spain more than 300 half-marathon races are held annually, with an important number of runners. As a result, an increasing number of amateur runners train assiduously to finish the races and improve their personal race times. It is very helpful for them to know their ideal paces or speeds for training and competition. Accordingly, identifying these values in advance is an objective necessity, for both the athletes and their coaches.

The physiological variables related to performance have been previously described (Ramsbottom et al., 1987). In the case of long-distance runners, those variables obtained in the incremental laboratory tests, essentially maximal oxygen consumption (VO_2_max) and its related variables, have been very useful for observing the adaptations produced by training (Legaz Arrese et al., 2006), and to predict performance in competition (Foster, 1983). Additional laboratory tests and associated variables have been proposed that may be determinants of long-duration aerobic performance. Thus, the finish time in cross-country races has been associated with a high percentage of oxygen consumption (Loftin et al., 2007), as well as with blood lactate accumulation (Loftin et al., 2007), lactate threshold (Mujika et al., 2000), ventilatory threshold, or race economy (speed reached for a given oxygen consumption) (Amann et al., 2004; Saunders et al., 2004; Lucia et al., 2006; Støren et al., 2008; Santos-Concejero et al., 2017). However, it appears that the maximal speed achieved in the laboratory tests is the variable most closely associated with sports performance, regardless of the duration of the test (Noakes et al., 1990; Knechtle et al., 2011a; Ronconi and Alvero-Cruz, 2011).

Despite conclusions from previous research, most of the participants in these studies were elite or high-level athletes, and studies that focus specifically on the half-marathon are lacking (Tanaka et al., 1984; Weyand et al., 1994; Roecker et al., 1998). Those studies carried out in amateur runners described the following as the main predictors of final test performance: kilometers per week, number of weeks of training for each event, body mass index, resting heart rate (Campbell, 1985), anaerobic threshold and *v*VO_2_max (Roecker et al., 1998), anthropometric variables (Knechtle et al., 2011a, b, 2014), or fat percentage combined with average running speed during training (Knechtle et al., 2014). However, almost all the models used in these studies involve some form laboratory assessment, and are, therefore, hardly applicable in most of the amateur population.

The usefulness of field tests (Penry et al., 2011; Alvero-Cruz et al., 2017) for the assessment of the physiological construct of aerobic condition has shown great variability with respect to laboratory tests, although their validity in predicting the construct of final race performance has been poorly addressed in the literature. It should be noted that there is a paradox between the high reliability and low ecological validity of laboratory assessments and the low reliability and high validity of the methods used in field tests (Reilly et al., 2009; Galbraith et al., 2014; Llodio et al., 2016). The Cooper test, given its simplicity of application and low cost, has traditionally been widely used to estimate maximal oxygen consumption (Cooper, 1968), but its ability to predict constructs associated with sports performance in long-distance runners, such as half-marathon runners (Grant et al., 1995), has never been evaluated. Therefore, the objective of this study was to compare the predictive ability of half-marathon race time between two models derived from: (a) variables obtained in treadmill tests and (b) the Cooper test.

## Materials and Methods

### Experimental Approach to the Problem

During a single visit to the laboratory, various physiological performance variables were obtained through treadmill testing and anthropometric parameters were measured with the aim of predicting half-marathon finish time. It was hypothesized that a combination of physiological variables measured during physical effort would explain half-marathon performance. Prediction equations based on the laboratory variables that best predicted performance compared with the distance covered in the Cooper Test were subsequently developed.

### Participants

The laboratory study involved 23 amateur male athletes, with a mean age of 41.6 ± 7.4 years, with experience in training (8.3 ± 5.65 years) and long-distance races. The protocol was approved by the University Ethics Committee in accordance with the Declaration of Helsinki for human research. The participants were informed of the objectives, protocols and risks associated with the experiment, and signed a written informed consent to participate in the study.

### Experimental Design

**FIGURE 1:**
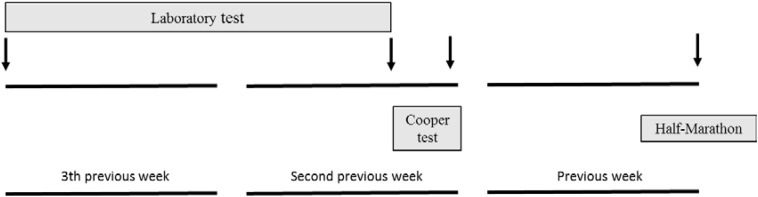
Schedule of laboratory and Cooper tests and the half-marathon.

Laboratory assessments were performed in February 2011, in a single session and the half-marathon race was held at the beginning of March of the same year. Between 10 and 21 days before the half-marathon a treadmill tests were carried out, and 7 to 10 days before the half-marathon race, in other session the athletes performed the Cooper test. All the athletes were engaged and motivated to perform at maximal effort, using their maximum heart rate for quality control of maximal effort exerted during the test (Figure 1).

For collection of the independent variables (associated with the different assessments), all the participants underwent a body composition assessment using anthropometry and an incremental exercise test in the laboratory with analysis of exhaled air, on the same day and within three weeks before the race. Subsequently, they undertook a Cooper test on a track, with an analysis of blood lactate. For each assessment, both in the laboratory and in the field test, which were conducted on different days, the participants were reminded to rest the day before and to have at least one meal with a high proportion of carbohydrates the previous day (rice and pasta).

### Procedures

#### Anthropometric Assessment

All measurements were conducted after a 12-h fast. Weight was measured on a SECA 813 electronic scale (Hamburg, Germany) with an accuracy of 0.1 kg, and height was measured using a wall mounted SECA 216 stadiometer (Hamburg, Germany) with an accuracy 0.1 cm. Skinfolds were measured in triplicate at the following sites: triceps, subscapular, biceps and iliac crest, computing the mean for their calculation. Percentage of body fat was estimated with the Durnin-Womersley equation (Durnin and Womersley, 1974). All measurements were collected under the standardized procedures of the International Society for Advancement in Kinanthropometry (Marfell-Jones et al., 2006). The technical error of measurement of the technician was less than 3% for skinfolds and 1% for the rest of the anthropometric measurements.

#### Laboratory Test

All participants underwent a maximum incremental exercise test to determine VO_2_max, as well as respiratory and metabolic exchange variables such as carbon dioxide output (VCO_2_), end-tidal oxygen tension (PetO_2_), end-tidal carbon dioxide tension (PetCO_2_), ventilation, and respiratory exchange ratio (RER). The expired gases were measured breath by breath and recorded in an Ultima gas analyzer system (MedGraphics, Saint Paul, MN, United States). The system was automatically calibrated before each test, according to the manufacturer’s instructions. Heart rate was recorded using a telemetric electrocardiography device (X-Scribe, Mortara, Milwaukee, WI, United States) connected to the system. Aerobic (VT1) and anaerobic (VT2) ventilatory thresholds were determined using Skinner and McLellan (1980) methodology. The participants ran on a treadmill (Ms Medisoft 870, Medisoft, Italy) according to the following protocol: After a 10-min warm-up at 5 km/h, the test began at 6 km/h with a constant gradient of 1%, and increases of 1 km/h/min until volitional exhaustion. The test was considered maximum when respiratory exchange ratio ≥ 1.15 or there was an increase of less than 2.1 ml/kg/min in VO_2_ between the two stages, or when a range ± 10 beats/min of the maximum predicted heart rate was reached, without these being excluding requirements according to ACSM Guidelines for exercise testing and prescription (American College of Sports Medicine, 2014). The velocity corresponding to VO_2_max (vVO_2_max), was defined as the minimum speed at which VO_2_max is reached (Billat et al., 1996). All the participants received verbal encouragement from the investigators to give their maximum possible effort. The percentage with respect to the theoretical heart rate (220-age) was calculated from the heart rate values. All tests were controlled by the research team.

#### Cooper Test

The Cooper test (Cooper, 1968) was performed on a 400-m synthetic athletic track with the supervision of the research team. Before the test began, a 15-min warm-up of continuous running was performed at a low-moderate pace in addition to calisthenics exercises. Subsequently, the participants carried out the classic test protocol, which consisted of covering the maximum possible distance for 12 min. Immediately after completion of the test, the distance traveled was measured by means of markers placed on the track at set intervals of 50 m. During the test, the participants used a 610 Polar monitor (Polar Electro Oy, Finland). The heart rate at the end of the Cooper test was considered the maximal heart rate achieved in the test. Additionally the perceived effort of the trial was recorded at the end of the test using the modified Borg visual scale (Borg, 1982).

#### Blood Lactate

At the end of the Cooper test and in the first minute (Cabral-Santos et al., 2017), a 0.5-μl blood sample was extracted from the earlobe for measurement of the blood lactate concentration using an enzyme electrode method (Lactate Pro LT-1710, Arkray, Japan). The objective of this analysis was to corroborate the lactate level after a steady state pace. The coefficient of variation of the analyzer used is 3%.

#### Weather Conditions

The weather conditions on the day of the half-marathon were cloudy, with wet soil but no rain at the time of the test. Testing took place between 10:00 a.m and 12:00 p.m, and temperatures ranged from 14 to 17°C with a relative humidity of 42% and wind less than 14 km/h.

### Statistical Analyses

The data are presented as means and standard deviations. Normality was analyzed using the Shapiro-Wilk test. Since all the variables were normally distributed, an association analysis between variables was performed using the Pearson correlation coefficients. Variables significantly associated with race time in the half-marathon were included in a stepwise multiple regression analysis to estimate the predictors of race time (dependent variable) from two blocks of independent variables (from the laboratory and the Cooper test). Subsequently, a concordance analysis was carried out between the values predicted with the equations, obtained from the Cooper test and the laboratory model, and the actual race times in the half marathon, using Bland-Altman procedures (Bland and Altman, 1986). The difference between the values was tested with a Student’s t-test and the bias using Kendall’s tau correlation coefficient. The level of significance in all cases was set at p < 0.05. The statistical analysis was performed on MedCalc Statistical Software version 19.1 (MedCalc Software bv, Ostend, Belgium, 2019)^1^.

## Results

The characteristics of the participants are shown in Table 1. All were older than 30 years of age, and their body mass index values and fat mass percentage indicated that they did not have excess adiposity.

**TABLE 1:** Characteristics of the sample (*n* = 23).

	**Mean ± *SD***
Age (years)	41.66 ± 7.46
Weight (kg)	70.38 ± 8.15
Height (cm)	172.54 ± 6.35
Body mass index (kg/m^2^)	23.60 ± 1.99
Fat mass (%)	15.73 ± 4.68

Table 2 presents the data on the laboratory variables. The mean HR max at the end of the treadmill test was 181 ± 14 bpm and the heart rate of the Cooper test of 177 ± 13 bpm without significant differences (*P* = 0.47). Respiratory exchange ratio values at the end of exercise confirm maximal effort.

**TABLE 2:** Treadmill test variables.

**Variable**	**Mean ± *SD***
VO_2_VT1 (mL/kg/min)	36.48 ± 5.77
VO_2_VT2 (mL/kg/min)	48.63 ± 7.24
VO_2_max (mL/kg/min)	55.73 ± 8.34
HRVT1 (ppm)	140.81 ± 14.60
HRVT2 (ppm)	165.28 ± 15.07
HRMax (ppm)	180.63 ± 14.74
HRMaxLab/HRMaxTheo (%)	101.34 ± 8.60
VelVT1 (km/h)	11.16 ± 1.20
VelVT2 (km/h)	15.31 ± 1.88
*v*VO_2_max (km/h)	18.43 ± 1.80
RERVT1	0.85 ± 0.07
RERVT2	0.99 ± 0.08
RERMax	1.15 ± 0.11

*VO_2_VT1, oxygen uptake at VT1; VO_2_VT2, oxygen uptake at VT2; HRVT1, heart rate at VT1; HRVT2, heart rate at VT2; HRMax, maximum heart rate; HRMaxLab/HRMaxTheo, percentage MHRLab/MHR theoretical; VelVT1, speed at VT1; VelVT2, speed at VT2; *v*VO2max, speed at maximal oxygen uptake; RERVT1, respiratory exchange ratio at VT1; RERVT2, respiratory exchange ratio at VT2; RERMax, maximal respiratory exchange ratio.*

### Cooper and Blood Lactate Test

The Cooper test values generally denote a maximal effort test in relation to mean lactate values of 8.31 ± 2.87 mmol/L and the high percentage of maximum theoretical heart rate (Table 3).

**TABLE 3:** Test de Cooper variables.

**Variable**	**Mean ± *SD***
Cooper distance (m)	3121.48 ± 320.04
HR Cooper (bpm)	177.93 ± 13.56
HR Cooper/HRMax (%)	94.65 ± 23.37
Lactate after Cooper (mmol/L)	8.31 ± 2.87

*HR, heart rate; HRMax, maximum heart rate (220-age).*

### Half Marathon Race Time

The half-marathon race was completed by all 23 runners. The mean race time of the runners was 93.28 ± 10.28 min, range (73–117 min), (CV = 11%), at an average speed of 13.68 ± 1.57 km/h (CV = 11%).

### Bivariate Correlations

Table 4 shows the correlation coefficients between the half- marathon race time and the different variables. Of note are the correlations with distance covered in the Cooper test (*P* < 0.0001), velocity at ventilatory threshold 2 (vVT2), vVO2max (both *P* < 0.0001), VO_2_VT2 (*P* < 0.0001), and VO_2_max (*P* < 0.0005).

**TABLE 4:** Pearson correlation coefficients between half marathon race time and treadmill variables.

**Variable**	***r***	***P***
HRVT1	–0.058	0.78
HRVT2	–0.215	0.3
HRMax	–0.025	0.9
*v*VT1	–0.361	0.07
*v*VT2	–0.723	< 0.0001
*vVO_2_m*ax	–0.849	< 0.0001
VO_2_VT1	–0.292	0.15
VO_2_VT2	–0.79	< 0.0001
VO_2_max	–0.645	0.0005
Cooper distance	–0.932	< 0.0001

*HR, heart rate; *v*, speed; VT, ventilatory threshold; *v*VO_2_max, speed at VO_2_max; *r*, correlation coefficient.*

### Multiple Regression Analysis

Table 5 shows the two half-marathon time prediction models. In the first model, the variable of the distance covered in the Cooper test with the equation: Race time (min) = 201.26 – 0.03433 (Distance covered Cooper test) and in the model derived from the treadmill assessment, the maximum speed reached in the test together with the body weight are of note with the next equation:

Racetime(min)=156.7177-4:7194(vVOm2ax)+0.3435(Weight).

**TABLE 5:** Multiple regression models derived from field and laboratory tests.

**Model**	**Dep. variable**	**Indep. variable**	**Coefficient**	***R*^2^**	***R*^2^ adj**	**MCC**	**SEE**	**t**	***P***	**VIF**
Cooper test	Race time (min)			0.873	0.866	0.934	3.78			
		Constant	201.26							
		Distance covered	–0.03433					–11	< 0.0001	1
		Cooper test								
Treadmill test	Race time (min)			0.769	0.75	0.877	5.28			
		Constant	156.7117							
		*v*VO_2_max	–4.7194					–7.9	< 0.0001	1.5
		Weight	0.3435					2.25	0.0339	1.05

**R^2^, coefficient of determination; adj, adjusted; MCC, multiple correlation coefficient; SEE, standard error of the estimate; VIF, variance inflation factor.**

### Concordance Analysis

**FIGURE 2:**
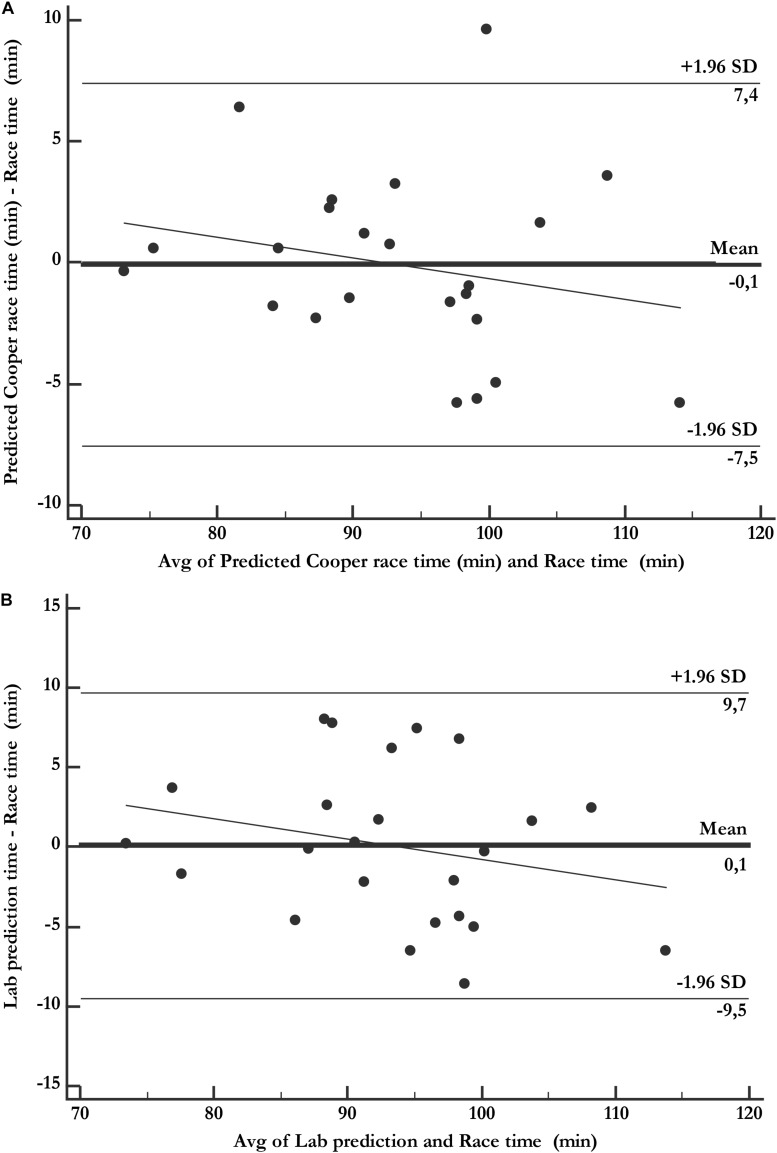
Bland-Altman plots comparing predicted time with actual race time. **(A)** Cooper test. **(B)** Laboratory test.

The differences between the predicted value (Cooper test model) and the actual time in the half marathon were not significant (dif = −0.08 ± 3.8 min, *P* = 0.91), or bias (Kendall’s tau, *r* = −0.18) (*P* = 0.40), with concordance limits of −7.5 to 7.4 min (Figure 2A). The laboratory test model values also showed no significant differences with the actual time (diff: −0.17 ± 5.03) (*P* = 0.83), with concordance limits of −9.7 to 10.0 min (Figure 2B).

The coefficient of variation between performance subjects (CV% = 100 × SD/mean) was calculated and the mean difference between the Cooper test was 20.46 m (95% CI: −20.22–61.15) (Alvero-Cruz et al., 2017).

## Discussion

To the best of our knowledge, this is the only study that has evaluated and compared the ability to predict race time in the half-marathon by means of a graded exercise test in the laboratory and a Cooper test. In this work, we have identified variables associated with race time in the half-marathon through both laboratory assessment and the Cooper test. In the literature, there were several studies on different performance determinants in middle- and long-distance runners, such as anthropometric variables (Arrese and Ostáriz, 2006; Knechtle et al., 2010, 2014; Friedrich et al., 2014), variables related to training load (Ramsbottom et al., 1987; Knechtle et al., 2011a; Balsalobre-Fernández et al., 2014) as well as physiological variables (Roecker et al., 1998; Reilly et al., 2009; Rabadán et al., 2011; Ronconi and Alvero-Cruz, 2011; Gómez-Molina et al., 2017; Støa et al., 2010), which have associations with half-marathon performance.

Our results reveal that the variables from both the laboratory and the Cooper test enable significant models to be obtained for the prediction of half-marathon race time. In addition, we found that the regression model from the distance covered in the Cooper test has a better predictive value. According to results of this study, the model developed from the distance covered in the Cooper test, would explain 87.3% of the total variance of half-marathon race time in recreational runners. The laboratory model that combines *v*VO2max and body weight would explain 76.95% of the total variance. The variables included in our models find partial similarities with other published studies. With respect to anthropometric variables, the skinfolds of the lower limbs, anterior thigh and medial calf are associated with performance in runners of 1500 and 10000 m (Arrese and Ostáriz, 2006). Rüst et al. (2011) found that race time in the half marathon in amateur runners was associated with BMI and average training speed. Similar results are also found in relation to BMI (Friedrich et al., 2014) and sum of six skinfolds with race time in half-marathon (Gómez-Molina et al., 2017) in male runners. Other authors have linked the fat mass percentage with performance in half- marathon (Knechtle et al., 2014).

The predictive model of this author explains only 44% of the race time, which can be explained by the wide limits of concordance in the prediction of race time (±25 min) and the great heterogeneity of the study sample. In our study, the only anthropometric variable that predicts race time is body weight.

Training load variables have traditionally been associated with performance in the half marathon. These include average training speed, previous experience, distance per week, hours per week, and daily training time (Friedrich et al., 2014). In the last of these studies, associations were found with the average training speed (*r* = −0.77 and *r* = −0.58, for women and men, respectively), with fat mass percentage and the average training speed as predictors. Despite the previous evidence, no training variables were introduced into our models, which must indicate the strong dependence of training on the variability of the test result.

The dependent variables in the laboratory prediction model explain 77% of the variance. *v*VO2max corresponds to around 72% and body weight to 5%. Similar aspects are those presented by Knechtle et al. (2014) who, in a recent study, found predictive models of race time in the half marathon based on body weight and average training speed as predictor variables, but explaining only 44% of the race time. The results of our research provide much more accurate equations, with a coefficient of determination of 0.873 for the equation derived from the Cooper test and 0.77 for the equation derived with body weight and *v*VO2max.

This group of researchers (Knechtle et al., 2014) sought to improve the prediction with new equations, introducing other independent variables into the model, such as the percentage of fat mass, obtaining an *R*^2^ of only 0.42 for the men and 0.68 for women. These coefficients are still low compared to those of our study. These authors also failed to measure VO_2_max and the maximum speed reached in the maximal graded test. They additionally analyze the accuracy of the prediction model using the limits of agreement (LA) between the actual value and the value predicted with the new equation. The LA of the study are very broad, between −25.6 and 24 min, with a proportional error as the race time increases. In our study, the LA for the equation derived from the distance covered in the Cooper test are from −7.4 to 7.5 min with no proportional error (*P* < 0.05).

The laboratory tests allowed us to obtain multiple variables, all of them well controlled and generally very reproducible. Maximal oxygen uptake is normally a good predictor of performance in long-distance runners (Hagan et al., 1981). However, this variable was not significant in either of our prediction models. Another factor that has frequently been associated with performance in runners is the maximum speed attained (*v*VO_2_max) in the incremental exercise test in the laboratory (Roecker et al., 1998). In our study, vVO2max was also a predictor in the laboratory model. This is likely due to race intensity in the half marathon being close to maximal oxygen consumption or maximum aerobic speed. Race time in the half marathon is not always explained by the absolute value of VO_2_max and is often best explained by the fractional use of VO_2_max, corresponding to a running speed, usually of a submaximal character and therefore to a submaximal VO_2_ value.

Nevertheless, Williams and Nute (1983) studied the physiological demands of half-marathon runners finding similarities in the physiological values of this study, in terms of the race times and VO_2_max values of the athletes. This study evaluated four runners and the variables that related to performance were VO_2_max and estimated speed at a concentration of 4 mmol/L of lactate, although they later verified that the average lactate values were 5.65 ± 1.42 mmol/L, which would confirm values close to VO_2_max.

Other studies such as that by Rabadán (Rabadán et al., 2011) analyze in the laboratory the physiological determinants of middle- and long-distance runners, finding that VO_2_max, VO_2_VT2 and *v*VT2 are variables that characterize these athletes. The strength of variables such as VT2 is that they are very reproducible parameters and therefore very useful in predicting and evaluating changes based on training. They can also help differentiate the performance of middle- and long-distance runners. In the present study, these values were not found to be predictors of performance.

### Limitations and Strengths of the Study

The main limitation is probably the small number of male participants and only performed in a half marathon event and therefore the results would only be applied to recreational runners between 73 and 117 min.

The main strength of this study is the comparison of prediction models in which the *v*VO_2_max is found as the most frequently variable related to performance in runners (Morgan et al., 1989; Noakes et al., 1990; Muñoz et al., 2012) and an easy-to-perform field test in a training schedule.

### Practical Applications

The number of long-distance runners has grown considerably in recent years. Accordingly, it is of great interest to trainers and sports science researchers to have accurate specifications regarding training and its intensities. This study enables the determination of the competition pace since an estimate of the final race time can provide greater precision in determining the different training paces, and can be assessed as often as necessary within the process. This also helps to guide the pace of the half-marathon race in amateur runners. The main application lies in the fact that a simple field test can substitute laboratory tests, offering more accurate information for the estimation of race performance, independently of physiological determinants. This undoubtedly provides a great advantage due to the simplicity of the procedure and the low economic and time cost.

## Conclusion

The present study described the predictive ability of finish time in the half-marathon races using variables obtained in the Cooper test and in laboratory evaluations, with a higher predictive ability found in the former. In addition to the Cooper test being statistically more powerful than the treadmill laboratory test, its main strength is that it does not require laboratory technology and can be introduced into the daily training routine to provide a relatively valid prediction of race time.

The high predictive power of distance covered in Cooper test suggest that athletes and coaches should give attention to control of training paces and as a tool for selecting adequate competition strategies in half-marathon. Altogether, these results may guarantee a high degree of applicability for predicting half-marathon time in recreational male runners for its great reproducibility.

## Data Availability Statement

The datasets generated for this study are available on request to the corresponding author.

## Ethics Statement

The studies involving human participants were reviewed and approved by the protocol used in this study was approved by the Ethics Committee of the University of Málaga (2013-EMEFYDE-005) and was in accordance with the Declaration of Helsinki. The patients/participants provided their written informed consent to participate in this study.

## Author Contributions

JA-C, MG, and EC conceived and designed the study. JA-C collected the data. JA-C, MG, EC, and FA analyzed and interpreted the data, and drafted the manuscript. JA-C, MG, EC, FA, PN, TR, and BK revised the manuscript and approved the final version.

## Conflict of Interest

The authors declare that the research was conducted in the absence of any commercial or financial relationships that could be construed as a potential conflict of interest.

## Footnotes

1 https://www.medcalc.org
